# Clinical features, laboratory characteristics and risk factors for mortality of COVID-19 patients in a secondary hospital in Oman during the first wave of the SARS-CoV-2 pandemic

**DOI:** 10.1186/s42269-022-00825-w

**Published:** 2022-05-16

**Authors:** Zayid K. Almayahi, A. V. Raveendran, Rashid Al Malki, Amira Safwat, Muradjan Al Baloshi, Amal Abbas, Ahmed S. Al Salami, Sami M. Al Mujaini, Khalid Al Dhuhli, Said Al Mandhari

**Affiliations:** 1 Disease Surveillance and Control Department, Ministry of Health, P.O. Box 543, P.C 329 Rustaq, South Batinah Governorate Oman; 2 Internal Medicine Department, Bader Al Samaa Hospital, Barka, Oman; 3 Laboratory Department, Rustaq Hospital, Ministry of Health, Rustaq, Oman; 4 Anesthesia Department, Rustaq Hospital, Ministry of Health, Rustaq, Oman

**Keywords:** COVID19, Oman, Mortality, Clinical characteristics, Laboratory, Risk factors, SARS CoV-2

## Abstract

**Background:**

The changing epidemiological profile of the COVID-19 pandemic and the uncertain clinical picture of patients characterise this ongoing and most challenging health event.

**Objectives:**

To report clinical features, laboratory characteristics, and mortality risk factors among COVID-19 patients admitted to a secondary hospital in Oman.

**Methods:**

A retrospective study for the first 455 patients admitted with COVID-19 to Rustaq hospital from 12th April, 2020 to 27th September, 2020. A predesigned questionnaire collected data from the hospital medical electronic system.

**Results:**

The mean age was 42.84 (SD = 19.86) years, and the majority of patients were aged 30 to 59 and 60 or above; 207 (45.5%) and 189 (41.5%), respectively. Male patients constituted approximately two-thirds of the subjects. Fever, dyspnea and cough were the most common presenting symptoms (69%, 66%, and 62%, respectively), while comorbidities with diabetes mellitus and hypertension were 47% and 44%, respectively. Bacterial growth was identified at approximately 10%. Bivariate analysis turned out to be significant with a number of factors. However, multivariate analysis showed significance with patients aged over 60 (OR = 7.15, 95% CI 1.99–25.63), dyspnea (OR = 2.83, 95% CI 1.5–5.33), dyslipidemia (OR = 1.93, 95% CI 1.02–3.66) and being bed-ridden (OR = 5.01, 95% CI 1.73–14.44). Durations from onset of symptoms to admission and respiratory distress were lower among patients who died; *p* = 0.024 and *p* = 0.001, respectively. Urea, Troponin and LDH may act as potential diagnostic biomarkers for severity or mortality.

**Conclusions:**

This study identified groups of patients with a higher risk of mortality, with severe disturbance in the laboratory markers while some could act as potential diagnostic biomarkers.

## Background

At the end of the year 2019, the world witnessed the emergence of COVID-19, a disease caused by severe acute respiratory syndrome coronavirus-2 (SARS-CoV2), in Wuhan, China (Zhu et al. 2020). COVID-19 is a highly contagious disease that rapidly infected many countries and was declared a pandemic on 11th March 2020 (Cucinotta and Vanelli 2020). By 27th October 2021, 244,385,444 people had been infected and 4,961,489 lives had been lost globally; Oman had 304,217 positive cases and 4,111 deaths (WHO Coronavirus Disease (COVID-19), 2020).

COVID-19 patients may present in a wide range of manifestations. However, according to estimates, 28–31% of infected patients are asymptomatic (Alene et al. 2021). Studies have also shown that disease severity correlates with several risk factors, especially old age, male gender and other comorbidities, such as hypertension, diabetes, chronic kidney disease, cancer and chronic liver disease (Gao et al. 2021). It was also found that increased age, the neutrophil-to-lymphocyte ratio (NLR) and white blood cells count were associated with higher COVID-19 mortality (Vafadar Moradi et al. 2021). A meta-analysis study, however, suggested to use serum levels of C-reactive protein, interleukin-6, and D-dimer as risk stratification tools to identify Covid-19 patients with poor clinical outcomes (Suri et al. 2022). The mortality rate can also double when patients develop hospital-acquired infections (HIAs) due to prolonged mechanical ventilation and hospitalisation and the increased likelihood of septic shock (Grasselli et al. 2021).

The changing clinical as well as epidemiological characteristics of the Covid-19 virus has been reported too. In an Italian study, the proportion of patients with symptomatic infections and the need for hospitalisation among fully vaccinated individuals was higher among those infected with Delta variant compared to Alpha variant; Delta group showed odds of 3.08 (95% CI 2.55–3.72), and 2.66 (95% CI 1.76–3.94), for symptomatic infections and hospitalization, respectively (Loconsole et al. 2021). At the same time, it remains challenging to certainly know the long-lasting post Covid-19 consequences. The need for oxygen therapy, pre-existing hypertension, chronic pulmonary disorders, and any comorbidity were found as the main determinants of the persistent post-Covid-19 symptoms, in a study from Egypt (Galal et al. 2021).

This report adds the experience of the South Batinah Governorate (SBG) to the existing literature. The SBG, which is the fourth most populated governorate in Oman, was one of the first governorates to be hit by the COVID-19 pandemic during the study period. The study explores the initial presentation, time course of symptoms, admission and discharge, clinical characteristics and available laboratory parameters for the first 455 patients admitted to the governorate’s secondary hospital. Additionally, the study compares different variables with the survival and mortality outcomes and suggests possible predictors of the likelihood of death.

## Methods

The SBG is the fourth most populated governorate in Oman and has 466,424 inhabitants (eCensus Portal 2021). It is divided into six wilayats (districts) with 21 primary health institutions and two preventive medical centres. The SBG is served by a 236-bed secondary hospital in the Rustaq district. An isolation ward was designated for COVID-19 cases, and its capacity was upgraded based on the epidemiological situation. This study included all patients who tested positive by real-time polymerase chain reaction test (RT–PCR) and who were admitted to the Rustaq hospital from the first case on 12th April 2020 up to 27th September 2020. The total number of patients amounted to 455.

### Data collection

Patients’ data underwent retrieval from the hospital’s electronic medical system known as Al Shifa (a comprehensive electronic healthcare information management system developed by the Ministry of Health-Oman). Details of the COVID-19 test were obtained from another surveillance platform of the Ministry of Health: Tarassud, which is an electronic notification system of communicable diseases. The information was then added into an Excel electronic questionnaire categorised in the following sections. The first section included demographic information and details on the dates of admission, the onset of symptoms, collection and release dates of the COVID-19 test, dates of respiratory distress, intervention and outcome. The second section focused on data recording symptoms, comorbidities and vital signs on admission. The third section focused on laboratory and radiographic information, the medications and their durations. The collection of data required comprehensive searches of the patients’ electronic files and a solid understanding of the clinical information and status of the patients. Therefore, in this research, four medical staffs were trained to collect the data, supervised by a co-author medical doctor.

### Laboratory testing

The hospital followed the case definitions approved by the MOH: a suspected case of Covid-19 was defined as a person with the acute respiratory syndrome (which included the sudden onset of at least one of the following symptoms: fever ≥ 38 °C, cough, shortness of breath, sore throat) and/or a person with pneumonia and/or a patient admitted with a severe acute respiratory infection (SARI) or one who developed a SARI in hospital.

Nasopharyngeal and oropharyngeal swabs were collected and kept in containers filled with a viral transport medium (VTM) solution. All swabs were sent to central public health laboratories (CPHL) for real-time PCR before April 21st 2020. After that, a new system to test for SARS COV-2 was installed in Rustaq Hospital Laboratory. It is a GeneXpert system XVI-8 which works as a second-generation molecular PCR programme. This system is fully automated and uses a consumable cartridge containing all required reagents to run a single sample test. It was developed to detect the E gene and the N2 gene specific to the SARS COV-2 virus. Numerous studies compared this system with conventional RT PCR and reported a pronounced concordance (Vaz et al. 2021).

As for the suspicion criteria to screen for bacteria, there was no written protocol. However, patients were screened based on clinical judgment which basically included admission to intensive care unit, increased in white blood cells and unexpected worsening in the clinical condition. Blood sample was collected into BD aerobic and anaerobic blood culture bottles using sterile blood collection steps. Samples were incubated inside BD BACTEC Instrumented blood culture system machine for 24 to 120 h and if the bacterial growth was detected, the samples were processed by manual rapid bacterial identification and susceptibility testing system (Advancing the world of health | BD 2021). The DxC Beckman Coulter 700 AU was used to run the biochemistry analysis (Beckman Coulter Diagnostics 2021).

### Statistical analysis

The IBM SPSS 23.0 (IBM CorpArmonk, New York USA) programme organised, tabulated and analysed the data. The numerical information was presented as mean and standard deviation, while the categorical information was presented as numbers and percentages. The student t-test found the mean differences of the laboratory parameters between discharged and dead patients. A Receiver Operating Characteristic (ROC) curve in SPSS identified lab parameters to help predict in-hospital mortality and their cut-off values. Kaplan–Meier survival curves compared the survival rate for patients by the log-rank test. Odds ratios (OR) and related 95% confidence intervals (95% CI) were calculated using bivariate and multivariable analyses (unconditional binary logistic regression). Only statistically significant covariates in the bivariate analyses formed part of the multivariable model. The *p* value adopted was *p* ≤ 0.05.

### Ethical consideration

The study received ethical approval from the Research and Ethical Review & Approve Committee at SBG on 13th July 2020. The research code is 01072020. The personal data, medical investigations and outcomes underwent anonymous collection and analysis.

## Results

This analysis included 455 patients admitted from 12th April 2020 to 27th September 2020, of which 112 (24.6%) have died. The majority of patients, 207 (45.5%), were aged 30 to 59, compared to 189 (41.5%) aged over 60, with a mean age of 52.84 (SD = 19.86). Approximately two-thirds of admitted patients were males, and the predominant nationality was Omani; 409 (90%). The study featured 275 (60.4%) patients admitted for a duration less than six days, with an average stay of 7.32 days (SD = 10.54); 85 (18.7%) patients were intubated. Fever, dyspnea and cough were the three most common presenting symptoms (69%, 66% and 62%, respectively). They were followed by gastrointestinal symptoms (24%), bodyache (16%) and fatigue (11%), while only approximately 5% of patients experienced sore throat and dizziness. Less than 4% had headache, ageusia, insomnia and runny nose. Diabetes mellitus (DM) and hypertension were the most common comorbidities (47% and 44%, respectively), followed by cardiac diseases (20%) and dyslipidemia (16%). Other conditions, including respiratory, neurological, renal, haematological and malignancy, accounted for less than 10%. Additionally, 5.7% of patients were bed-ridden. Out of 221 patients who had their dates of onset available, approximately 58% were admitted within five days or less after they developed symptoms. Of the 147 patients who had growth culture tests, 47 came back positive. Coagulase-negative *Staphylococcus* was the predominant bacterial strain, representing approximately 23%, followed by *Pseudomonas aeruginosa* (10%), *Acinetobacter Baumannii* (10%) and *Klebsiella Pneumonia* (8%).

The bivariate analysis was positive for numerous factors, including age, dyspnea, hypertension, dyslipidemia, being bed-bound, renal diseases and positive culture growth. Taking patients aged less than 30 as a reference, the likelihood of death had significantly increased; (OR = 3.80, 95% CI 1.12–12.83) and (OR = 12.01, 95% CI 3.63–39.79) among patients aged 30 to 59 and 60 or above, respectively. In terms of symptoms, only dyspnea had a significant association, showing odds ratios of 2.66, 95% CI 1.59–4.44, compared to those with no breathing issues. Similarly, the death likelihood increased for those with hypertension (OR = 2.05, 95% CI 1.33–3.17), dyslipidemia (OR = 2.31, 95% CI 1.37–3.92), cardiac diseases (OR = 1.75, 95% CI 1.05–2.92), renal diseases (OR = 2.24, 95% CI 1.07–4.70) and those who are bed-bound (OR = 5.55, 95% CI 2.44–12.63). However, with regards to the multivariate analysis, the following factors demonstrated a significant association: being aged over 60 (OR = 7.31, 95% CI 2.08–25.70), dyspnea (OR = 2.91, 95% CI 1.65–5.12), dyslipidemia (OR = 1.93, 95% CI 1.02–3.66); 0.044), and being bed-ridden (OR = 3.49, 95% CI 1.42–8.57) (Tables 1 and 2).

**Table 1:** Patient characteristics, unadjusted and adjusted odds ratios and 95% confidence intervals for the likelihood of death

	All patients	Discharged	Death	Unadjusted OR (95% CI); *p* value	Adjusted OR (95% CI); *p* value
	455	343	112		
Age					
< 30 years	13%, 59/455	56, 16.3%	3, 2.7%	Reference	Reference
30–59 years	45.5%, 207/455	172, 50.1%	35, 31.3%	**3.80 (1.13–12.83);0.032**	2.5 (0.71–8.82); 0.153
≥ 60 years	41.5%, 189/455	115, 33.5%	74, 66.1%	**12.01 (3.63–39.79); <0.001**	**7.15 (1.99–25.63); 0.003**
Nationality					
Omani	89.9%, 409/455	308, 89.9%	101, 90.2%	1.04 (0.511–2.130); 0.907	N/A
Non-Omani	10.1%, 46/455	35, 76.1%	11, 9.8%	Reference	N/A
Gender					
Male	65.9%, 300/455	233, 65%	77, 68.8%	1.18 (0.75–1.87); 0.469	1.52 (0.89–2.61); 0.129
Female	34.1%, 155/455	120, 77.4%	35, 31.3%	Reference	Reference
Symptoms					
Fever	69%, 314/455	234, 68.2%	80, 71.4%	1.17 (0.73–1.86); 0.524	1.17 (0.68–2.01); 0.583
Dyspnea	65.5%, 298/455	208, 60.6%	90, 80.4%	**2.66 (1.59–4.44); < 0.001**	**2.83 (1.5–5.33); 0.001**
Cough	62.2%, 314/455	90, 60.9%	74, 66.1%	1.25 (0.80–1.95); 0.331	0.89 (0.51–1.52); 0.66
GI Symptoms	23.5%, 107/455	86, 25.1%	21, 18.8%	0.69 (0.40–1.18); 0.172	0.94 (0.51–1.73); 0.84
Bodyache	16.3%, 74/455	54, 15.7%	20, 17.9%	1.16 (0.66–2.05); 0.599	1.11 (0.55–2.22); 0.772
Fatigue	11.4%, 52/455	40, 11.7%	12, 10.7%	0.91 (0.46–1.80); 0.784	1.07 (0.47–2.45); 0.877
Sore throat	5.5%, 25/455	21, 6.1%	4, 3.6%	0.57 (0.19–1.69); 0.310	1.2 (0.36–4.06); 0.768
Dizziness	5.1%, 23/455	17, 5.0%	6, 5.4%	1.09 (0.42–2.82); 0.867	1.36 (0.46–4.02); 0.576
Headache	3.5%, 16/455	14, 4.1%	2, 1.8%	0.43 (0.01–1.91); 0.266	0.44 (0.09–2.21); 0.317
Ageusia	1.5%, 7/455	7, 2.0%	0	0.199 (0.011–3.52); 0.271	0; 0.999
Inosmia	1.3%, 6/455	6, 1.7%	0	0.23 (0.013–4.13); 0.319	0; 0.999
Runny nose	0.7%, 3/455	3, 0.9%	0	0.43 (0.02–8.44); 0.580	0; 0.999
Comorbidities					
HTN	43.7%, 199/455	135, 39.4%	64, 57.1%	**2.05 (1.33–3.17); 0.001**	0.88 (0.5–1.56); 0.673
DM	46.6%, 212/455	151, 44%	61, 54.5%	1.52 (0.99–2.33); 0.055	1.13 (0.68–1.87); 0.649
DLP	16.3%, 74/455	45, 13.1%	29, 25.9%	**2.31 (1.37–3.92); 0.002**	**1.93** **(1.02–3.66); 0.044**
Respiratory	6.4%, 29/455	19, 5.5%	10, 8.9%	1.67 (0.75–3.71); 0.207	1.66 (0.66–4.17); 0.284
Cardiac	18.9%, 86/455	57, 16.6%	29, 25.9%	**1.75 (1.05–2.92); 0.031**	0.822 (0.43–1.56); 0.547
Hematological	3.1%, 14/455	11, 3.2%	3, 2.7%	0.83 (0.23–3.03); 0.779	1.577 (0.36–6.78); 0.549
Neurological	9.0%, 41/455	31, 9.0%	10, 8.9%	0.99 (0.47–2.08); 0.99	0.531 (0.204–1.38); 0.194
Bed ridden	5.7%, 26/455	10, 2.9%	16, 14.3%	**5.55 (2.44–12.63); < 0.001**	**5.01 (1.73–14.44); 0.003**
Renal prob	7.0%, 32/455	19, 5.5%	13, 11.6%	**2.24 (1.07–4.70); 0.033**	1.84 (0.78–4.37); 0.167
Malignancy	1.8%, 8/455	4, 1.2%	4, 3.6%	3.14 (0.77–12.76)	1.99 (0.42–9.32); 0.385
Bacterial growth					
Growth	32%, 47/147	9, 11%	38, 58.5%	**11.42 (4.88–26.71); < 0.001**	N/A
No growth	68%, 100/147	73, 89%	27, 41.5%	Reference	N/A
Duration form onset to admission					
≤ 5 days	57.7%, 128/222	92, 54.8%	36, 66.7%	Reference	N/A
≥ 6 days	42.3%, 94/222	76, 45.2%	18, 33.3%	0.61 (0.32–1.15); 0.125	N/A
G6PD					
Deficiency	26.1, 18/69	16, 30.8%	2, 11.8%	0.3 (0.06–1.47); 0.137	N/A
Normal activity	73.9, 51/69	36, 69.2%	15, 88.2%	Reference	N/A
Hospital stay	Mean (SD): 7.32 (10.54), IQ (2–9)				
≤ 6 days	275 (60.4%)				
≥ 6 days	180 (39.5%)				
Intubated patients	85)18.7%)				

Bold indicates statistically significant

**Table 2:** Positive culture growths of 47 patients hospitalised with COVID19

	Blood	ET tube	Others^a^	Total
*Coagulase-negative Staphylococcus*	20	0	0	20
*Enterococcus Faecium*	3	0	1	4
*Gram-negative bacilli*	2	0	0	2
*Gram-positive bacilli*	1	0	0	1
*Klebsiella Pneumoniae*	3	4	0	7
*MDR Acinetobacter*	1	2	1	4
*Methicillin-resistant Staphylococcus Aureus*	2	0	2	4
*Pseudomonas Aeruginosa*	3	6	0	9
*ESBL-producing E coli*	2	1	0	3
*Stenotrophomonas Maltophilia*	0	3	0	3
*Acinetobacter Baumannii*	0	8	1	9
*Burkholderia Pseudomallei*	0	1	0	1
*E coli*	0	1	0	1
*Candida (albicans, tropicalis, rugosa, *etc*.)*	2	8	1	11
*Streptococcus (Pyogenes, Group D, *etc*.)*	4	1	0	5
*Staphylococcus (Aureus, *etc*.)*	1	2	0	3
*Total*	44	37	6	87

Statistically significant was shown in bold

^a^Sputum, throat, urine and rectal

Of the 23 laboratory tests that compared survivors and non-survivors, 15 tests showed significant differences. For example, patients who died had increased white blood cell (WBC) and neutrophil counts, C-reactive protein (CRP), Urea, Lactate dehydrogenase (LDH) and Alanine aminotransferase (ALT) compared to patients who were discharged (*p* < 0.001). The same holds true for other laboratory parameters, including Creatinine (*p* = 0.003), Troponin (*p* = 0.002) and Ferritin (*p* = 0.021). Among the respiratory system function indicators, the mean of PCO2 and FiO2 proved to be higher among those who died compared to the discharged patients; *p* = 0.032 and < 0.001, respectively. The discharged patients, however, had higher SpO2 and lower respiratory rates on admission (*p* < 0.001 and *p* = 0.006, respectively). PaO2/FiO2 was also higher among the discharged patients (*p* = 0.001). The duration between the onset of symptoms and the admission and respiratory distress dates were lower among those who died: *p* = 0.024 and *p* = 0.001, respectively (Table 3).

**Table 3:** Comparison of Laboratory parameters, vital signs and respiratory function indicators and outcome of hospitalised patients with COVID19

Lab test, units	Normal range	Death		Discharged		*p* value
		*N*, %	Mean + SD	*N*, %	Mean + SD	
WBC count, 10^9^/L	4–11	112, 24.8	9.54 ± 6.77	340, 75.2	7.11 ± 3.77	< 0.001*
Neutrophils, per cmm	2–7.5	112, 24.8	7.77 ± 6.31	340, 75.2	5.20 ± 3.46	< 0.001*
Lymphocytes, per cmm	1.5–4.5	112, 24.8	1.13 ± 0.71	340, 75.2	1.30 ± 0.77	0.039*
Eosinophils, per cmm	0.0–0.4	112, 24.8	0.02 ± 0.08	340, 75.2	0.03 ± 0.07	0.232
Basophils, per cmm	0.0–0.1	112, 24.9	0.05 ± 0.05	338, 75.1	0.06 ± 0.14	0.525
Platelet count, 10^3^/L	150–450	112, 24.8	225.43 ± 105.94	340, 75.2	234.08 ± 100.07	0.434
Hemoglobin, g/dL	11.5–16.5	112, 24.8	12.25 ± 2.51	340, 75.2	12.67 ± 2.06	0.074
CRP, mg/L	0–5	106, 25.3	153.70 ± 97.73	313, 74.7	110.15 ± 88.64	< 0.001*
Serum Urea, mmol/L	2.5–7.5	112, 25.7	19.80 ± 14.04	324, 74.3	6.97 ± 6.21	< 0.001*
Serum sodium, mmol/L	136–146	112, 25.7	137.18 ± 9.27	324, 74.3	135.10 ± 4.42	0.002*
Serum creatinine, µg/L	45–100	112, 25.7	147.66 ± 190.67	324, 74.3	98.54 ± 132.10	0.003*
eGFR, ml/min/1.73m^2^	> 90	111, 26.6	44.53 ± 31.25	306, 73.4	77.88 ± 21.75	< 0.001*
Troponin, pg/ml	0–14	94, 29.4	253.48 ± 955.45	226, 70.6	45.54 ± 167.142	0.002*
LDH, U/L	240–480	105, 29.9	1042.17 ± 2418.38	246, 70.1	429.58 ± 18,503	< 0.001*
Ferritin, µg/L	18–323	102, 29.6	2572.26 ± 5558.68	243, 70.4	1532.26 ± 2763.93	0.021*
Glucose, mmol/L	< 11	69, 31.5	15.27 ± 6.45	150, 68.5	13.09 ± 7.06	< 0.030*
D-dimer, µg/mL	0–0.5	17, 19.1	2.02 ± 2.17	72, 80.9	2.16 ± 6.97	0.934
Serum lactate, mmol/L	0.5–2.2	27, 51.9	2.15 ± 0.91	25, 48.1	1.83 ± 0.901	0.210
INR	2–4	60, 34.7	1.25 ± 0.302	113, 65.3	1.43 ± 1.92	0.457
Corrected calcium,mmol/L	2.1–2.6	59, 41.5	2.26 ± 0.14	83, 58.5	2.27 ± 0.12	0.869
ALT, IU/L	0–40	109, 26.2	214.90 ± 767.43	307, 73.8	52.61 ± 82.81	< 0.001*
Total bilirubin, mmol/L	3–17	109, 26.2	17.03 ± 16.95	307, 73.8	13.49 ± 15.84	0.049*
Albumin, g/L	35–50	109, 26.1	28.51 ± 5.18	308, 73.9	35.22 ± 17.22	< 0.001*
pH	7.35–7.45	76, 64.4	7.33 ± 0.122	42, 35.6	7.37 ± 0.104	0.062
pCO2, mmHg	35–45	76, 65.5	44.62 ± 21.05	40, 34.5	36.96 ± 9.76	0.032*
pO2, mmHg	80–100	74, 64.3	66.13 ± 30.12	41, 35.7	72.64 ± 40.13	0.328
HCO3, mmol/L	22–26	64, 68.1	23.28 ± 5.49	30, 31.9	24.32 ± 5.04	0.381
FiO2, %	–	86, 57.0	79.7 ± 23.77	65, 43.0	53.0 ± 23.06	< 0.001*
PaO2/FiO2	> 400	71, 65.7	89.53 ± 61.82	37, 34.3	140.61 ± 88.13	0.001*
Baseline QT interval, ms	< 430(M), < 450(F)	21, 22.6	449.90 ± 27.72	72, 77.4	446.38 ± 27.56	0.607
Temp, °C	–	96, 25.0	37.35 ± 0.92	288, 75.0	37.44 ± 0.93	0.458
SpO2, %	95–100	112, 24.6	81.23 ± 13.44	343, 75.4	90.98 ± 7.67	< 0.001*
sBP, mmHg	–	110, 25.1	130.89 ± 22.49	329, 74.9	131.25 ± 21.84	0.884
dBP, mmHg	–	110, 25.1	74.82 ± 14.60	328, 74.9	76.88 ± 12.35	0.149
Pulse, bpm	–	103, 26.5	96.2 ± 18.42	285, 73.5	96.06 ± 19.10	0.692
Respiratory rate, rpm	–	44, 29.1	29.39 ± 12.37	106, 70.9	25.00 ± 6.92	0.006*
Duration from symptoms onset to admission date	Days	54, 24.3	4.54 ± 2.951	168, 75.7	6.09 ± 4.74	0.024*
Duration from symptoms onset to respiratory distress	Days	54, 32.9	4.24 ± 3.18	110, 76.1	6.73 ± 5.00	0.001*

*Significant result

**Fig. 1:**
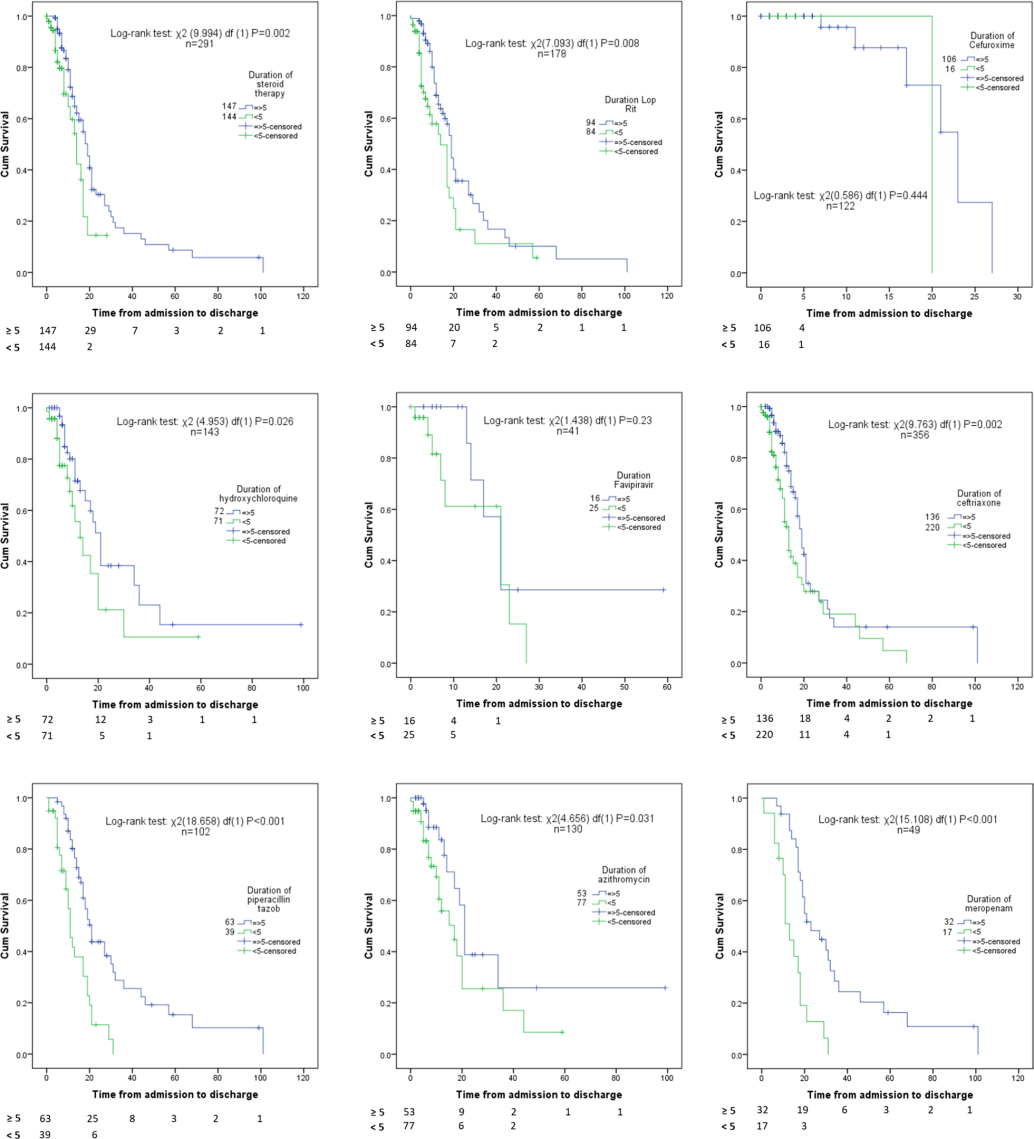
Kaplan–Meier survival curves of certain medications for hospitalised COVID19 patients

The Kaplan–Meier survival curves showed a significant lower survival rate in patients who received the following medications for less than five days: Steroidal therapy iv (*p* = 0.002), Hydroxychloroquine (*p* = 0.026), Piperacillin/Tazobactam (*p* < 0.001), Lopinavir/Ritonavir (*p* = 0.008), Azithromycin *p* = (0.031), Ceftriaxone (*p* = 0.002) and Meropenem (*p* < 0.001) (Fig. 1).

**Fig. 2:**
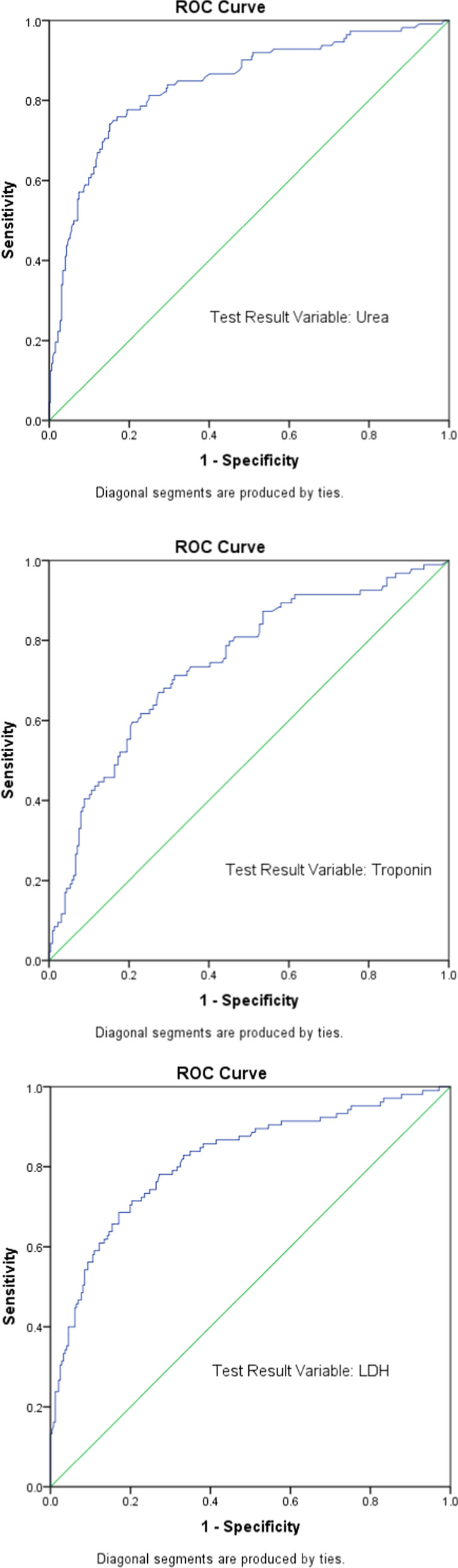
ROC curves used to suggest possible laboratory predictors for COVID 19 mortality

The areas under the ROC (AUC) of Urea, Troponin and LDH amounted to 0.841, 0.742 and 0.817, respectively, while the optimal cut-off values reached respective figures of 9.02, 21.15, and 514.25 (Fig. 2). Other laboratory parameters could not be used as a potential diagnostic biomarker for analysis because the AUC was less than 0.73 (Table 4).

**Table 4:** Areas under the ROC (AUC) for a number of laboratory parameters

Test Result Variable(s)	Missing	Area	Std. Error	Asymptotic Sig	Asymptotic 95% Confidence Interval	
					Lower Bound	Upper Bound
WBC	3	0.611	0.032	< 0.001	0.549	0.674
Platelet	3	0.463	0.032	0.245	0.400	0.527
Neutrophils	3	0.631	0.032	< 0.001	0.569	0.693
Lymphocytes	3	0.413	0.033	0.006	0.349	0.477
Eosinophils	3	0.421	0.030	0.012	0.362	0.479
Basophils	5	0.496	0.032	0.907	0.433	0.559
Hemoglobin	3	0.454	0.033	0.140	0.390	0.517
C- reactive protein	36	0.638	0.030	< 0.001	0.579	0.697
Urea	19	0.841	0.023	< 0.001	0.796	0.887
Sodium	19	0.573	0.037	0.022	0.500	0.646
Creatinine	19	0.639	0.031	< 0.001	0.579	0.700
eGFR	38	0.213	0.028	< 0.001	0.158	0.267
Troponin	135	0.742	0.031	< 0.001	0.682	0.803
LDH	104	0.817	0.026	< 0.001	0.766	0.868
Ferritin	110	0.615	0.033	0.001	0.551	0.678
Glucose	236	0.612	0.040	0.008	0.535	0.690
d-dimer	366	0.728	0.057	0.004	0.616	0.841
Lactate	403	0.625	0.080	0.122	0.469	0.782
INR	282	0.637	0.045	0.003	0.550	0.725
Corrected calcium	313	0.503	0.050	0.950	0.405	0.601
ALT	39	0.636	0.032	< 0.001	0.574	0.698
Total bilirubin	39	0.557	0.034	0.077	0.490	0.624
Albumin	38	0.212	0.024	< 0.001	0.164	0.259
NLR	3	0.665	0.031	< 0.001	0.605	0.725
PLR	3	0.562	0.034	0.050	0.495	0.628
LCR	36	0.654	0.031	< 0.001	0.593	0.715

	Area	Asymptotic Sig	Asymptotic 95% confidence interval		Sensitivity	1-Specificity	Cut-off value
			Lower Bound	Upper Bound			
Urea	0.841	< .001	0.796	0.887	0.777	0.192	9.015
Troponin	0.742	< .001	0.682	0.803	0.713	0.314	21.15
LDH	0.817	< .001	0.766	0.868	0.781	0.273	514.25

## Discussion

This study described the clinical profile and outcome of patients admitted with SARS-CoV-2 infection in one of the principal MOH hospitals in SBG in Oman. To the best of the researchers’ knowledge, this study is the first of its kind to focus on hospitalised patients infected with SARS-CoV2 in SBG, which represents one of the earliest governorates affected by COVID-19. The researchers reported a similar pattern of presentations among patients, with a short duration from onset until admission. A significant proportion of patients had other comorbidities, primarily DM and hypertension. However, mortality showed a marked association with certain comorbidities, but the most significant was old age and patients who had dyspnea or dyslipidemia or were bed-bound, with a pronounced disturbance in the laboratory parameters. Urea, LDH and troponin may act as potential predictors for COVID-19 mortality.

Two-thirds of admitted patients were males, similar to other studies showing male predominance (Wang et al. 2020; Gupta et al. 2020; Kumar et al. 2020; Huang et al. 2020a; Rivera-Izquierdo et al. 2020; Borghesi et al. 2021; Hasan et al. 2020; Chen et al. 2020; Guan et al. 2020; Grasselli et al. 2020; Grasselli et al. 2021). Various explanations for such circumstances include increased outdoor activity, higher levels of angiotensin-converting enzymes in males and a high density of immune-related genes and regulatory elements in X chromosomes extensively involved in immune responses (Schurz et al. 2019; Sama et al. 2020).

Fever, dyspnea and cough represented the three most common symptoms in the patients. Other studies have reported similar presentations. For instance, a tertiary care hospital in North India (Bhandari et al. 2020) reported cough (85.71%), fever (78.57%) and myalgia (64.28%), while fever (77%), cough (54%) and shortness of breath (20.8%) comprised the most common symptoms in another Indian study (Soni et al. 2021). Fever and cough were the most prevalent symptoms (46.3% and 29.5%, respectively) in another study from Oman (Balushi et al. 2020), where 7% of patients were hospitalised. The symptom profile depends upon the day of illness, stage of the disease and the characteristics of the study cohort, such as hospitalised patients, non-hospitalised patients and patients admitted in the critical care unit.

The majority of patients (58%) were admitted within five days of the onset of symptoms. Other studies revealed similar findings, wherein the mean time between the onset of symptoms and hospitalisation amounted to less than four days (Feng et al. 2009; Patel et al. 2021).

Common comorbidities seen in the patients include DM (47%), hypertension (44%), cardiac diseases (20%) and dyslipidemia (16%). A study from Oman reported DM (32%), hypertension (32%) and chronic heart and renal diseases (12.8%) as common comorbidities in their patient population (Khamis et al. 2020). Similarly, a study from Ethiopia revealed DM (48.9%), followed by hypertension (27%) and HIV/AIDS (17.6%) in their population (Sultan et al. 2021). The presence of co-morbidities depends upon the prevalence of various diseases in that geographical area and the predominant age group of patients affected with COVID-19, as the risk of hypertension and DM increases as people’s ages advance.

This study revealed the likelihood of increased risk of death among elderly people, patients who presented with dyspnea and those with hypertension, dyslipidemia, cardiac diseases and renal diseases, and were bed-ridden. However, dyspnea, bed-ridden status, dyslipidemia and over 60 of age only remained significant in multivariate assessments. Since it is a retrospective study, the researchers could not assess the importance of obesity in the patients because most medical records lacked data regarding body mass index (BMI). A similar study from Oman showed DM’s association with a higher mortality rate, whereas in this study’s cohort, the researchers did not find a significant association of mortality with DM (Khamis et al. 2020). A meta-analysis showed DM correlated with disease progression, increased severity and mortality with COVID-19 (Huang, Lim and Pranata 2020a, b). Potentially, the limited ability to defend microorganisms due to high blood glucose increases the risk of rapid progression of infections. The researchers believe that such a situation occurs because of a weak immune system and cytokine storm, and subsequent complications were less robust in people with poorly controlled DM. Additionally, the researchers hope that further well-structured studies will focus on this controversial aspect of the relationship between diabetes and COVID-19 mortality.

However, in line with this research’s findings, a substantial population-based study from Oman found that patients over 60 faced a higher risk of ICU admission and death (Al Wahaibi et al. 2021). Other comorbid conditions included BMI > 40, chronic kidney disease and chronic lung diseases (Al Wahaibi et al. 2021). Likewise, it was proved in previous studies that dyslipidemia is associated with higher severity and mortality of covid-19 (Atmosudigdo et al. 2021; Liu et al. 2021).

A previous study concluded that COVID-19 mortality can be attributed to the virus-activated “cytokine storm syndrome” or fulminant myocarditis (Ruan et al. 2020).

In this study, increased white blood cell and neutrophil counts, a rise in CRP, Urea, LDH, ALT, Creatinine, Troponin, total bilirubin and Ferritin bore a positive association with mortality. The high mean of PCO2 and FiO2 was also linked with increased risk of mortality. Other studies showed worse outcomes in COVID-19 patients with lymphocytopenia, hypoalbuminemia, elevated levels of aspartate aminotransferase (AST), ALT, total bilirubin, LDH, Creatinine and D-dimer (Chen et al. 2020; Feng et al. 2009; Huang et al. 2021). Lymphocytopenia occurs due to damage of the cytoplasmic component of the lymphocyte by SARS-CoV-2 viral particles, resulting in its necrosis or apoptosis (Gu et al. 2005). High values of inflammatory markers, such as CRP, correspond to poor prognosis (Khamis et al. 2020; Wang et al. 2020; Soni et al. 2021). LDH is secreted into the extracellular space when the cell membrane is damaged, and high LDH indicates lung injury in patients with COVID-19 pneumonia (Drent et al. 1996). In accordance with this study’s findings, other studies found that Troponin, Urea and LDH act as possible predictors for the severe outcome of COVID-19 infection (Hachim et al. 2020; Özyılmaz et al. 2020; Zhou et al. 2020; Huang et al. 2021; Zhang et al. 2021). Since the comparisons predicted mortality, the cut-off values in this study were markedly higher. Other laboratory parameters predicted the poor prognosis of COVID-19 infection, such as the Neutrophil‐to‐lymphocyte ratio (NLR), score based on the neutrophil, lymphocyte and platelet counts (NLP score), lymphocyte-to-CRP ratio (LCR), interleukin-6, D-dimer and high-sensitivity CRP (Ahmeidi et al. 2020; Lagunas-Rangel 2020; Zheng et al. 2020). Another study has also identified low Hb, procalcitonin (PCT) and CT score on admission as mortality predictors (Zhang et al. 2021).

Bacterial growth was identified in approximately 10% of the admitted patients. Among the positive cultures, the Coagulase-negative *Staphylococcus* represented the most predominant strain, followed by *Pseudomonas Aeruginosa* and *Acinetobacter Baumannii*. A high percentage of coagulase-negative *Staphylococcus*, primarily *Staphylococcus Epidermidis*, can result from contamination during the blood sampling process. A retrospective study showed that patients with COVID-19 have lower bacteremia rates than controls, and the contamination rate of blood culture is high (Sepulveda et al. 2020; Yu et al. 2020), which questions the routine use of antibiotics in people with COVID-19. Meanwhile, another study found no significant difference in antibiotic use between survivors and non-survivors (Zhang et al. 2021). Therefore, if this study excludes contamination, the findings would show that *Pseudomonas Aeruginosa* and *Acinetobacter Baumannii* are the most common causes of superinfection and the rate of bacterial infection is comparable with other international studies. An early Chinese study showed that the incidence of bacterial co-infection/superinfection was 7.7% (Zhang et al. 2020). Research in the UK involving more than 800 admitted patients found a low co-infection rate of approximately 3%, but superinfection amounted to more than 6% (Hughes et al. 2020). It also revealed that *Pseudomonas Aeruginosa* was the most common pathogen causing late-onset infection. Charles Feldman and Ronald Anderson published an extensive review about co-infection in patients with COVID-19. They concluded that patients with co-infection and superinfection have poorer outcomes (Feldman and Anderson 2021).

Although this study does not assess the therapeutic benefits of different medications, the significantly lower survival rate seen with various medications, such as intravenous steroids, indirectly indicates that these medications are usually administered to patients with severe or critical diseases. However, the survival rate was better among those treated for more than five days. A study showed the use of corticosteroid within the first seven of admission among patients hospitalised with COVID-19 pneumonia reduced mortality and decreased transfers to the ICU without an associated increase in bacteremia or fungemia (Ho et al. 2021).

This research has several limitations. Firstly, due to this being a retrospective study, the researchers could not obtain comprehensive data due to incomplete medical records. Secondly, being a hospital-based study, referral bias influenced the outcome. Thirdly, the small sample size could have affected the statistical significance of important parameters, and similarly for the incomplete data, especially for G6PD status. Fourthly, as there are other hospitals in the region, this study encompasses only a limited proportion of the population affected with COVID-19. Fifthly, patients transferred from the hospital to other tertiary care centres because of a lack of facilities or hospital beds, were not included in the study. Finally, this study is a single centre study, meaning the findings may not be generalised.

## Conclusions

The researchers present data for the first 455 admitted patients in a secondary hospital in SBG during the first wave of the pandemic. Fever, dyspnea and cough represented the most common presenting symptoms. Patients with hypertension, dyslipidemia, cardiac diseases and renal diseases had higher odds of COVID-19 mortality, whereas patients over 60, or with dyspnea or bed-ridden showed more pronounced risk factors for mortality on multivariate analysis. Moreover, numerous laboratory parameters correlate with bad prognosis, whereas Urea, LDH and troponin may be used to predict COVID-19 mortality. Further prospective studies should address the different characteristics of the infected patients and determine the best therapeutic modalities.

## Data Availability

The datasets used and/or analysed during the current study are available from the corresponding author on reasonable request.

## Abbreviations

ALT: Alanine aminotransferase
AST: Aspartate aminotransferase
BMI: Body mass index
CPHL: Central public health laboratories
CRP: C-reactive protein
DM: Diabetes mellitus
HIAs: Hospital-acquired infections
LCR: Lymphocyte to CRP ratio
LDH: Lactate dehydrogenase
NLP score: Neutrophil, lymphocyte and platelet counts
OR: Odds ratios
PCT: Procalcitonin
ROC: Receiver operating characteristic
RT-PCR: Real-time polymerase chain reaction
SARS-CoV-2: Severe acute respiratory syndrome coronavirus-2
SBG: South Batinah Governorate
SD: Standard deviation
VTM: Viral transport medium
WBC: White blood cell
